# Epidemiological and Clinical Characteristics of COVID-19: A Retrospective Multi-Center Study in Pakistan

**DOI:** 10.3389/fpubh.2021.644199

**Published:** 2021-04-14

**Authors:** Mehmood Ahmad, Bilal Mahmood Beg, Arfa Majeed, Sadaf Areej, Sualeha Riffat, Muhammad Adil Rasheed, Sammina Mahmood, Rana Muhammad Zahid Mushtaq, Mian Abdul Hafeez

**Affiliations:** ^1^Department of Pharmacology, Riphah International University, Lahore, Pakistan; ^2^Department of Pharmacology and Toxicology, University of Veterinary and Animal Sciences, Lahore, Pakistan; ^3^Department of Botany, Division of Science and Technology, University of Education, Lahore, Pakistan; ^4^Department of Parasitology, University of Veterinary and Animal Sciences, Lahore, Pakistan

**Keywords:** epidemiology, COVID-19, clinical characteristics, diagnosis, treatment, Pakistan

## Abstract

The emergence of a pathogen responsible for a mysterious respiratory disease was identified in China and later called a novel coronavirus. This disease was named COVID-19. The present study seeks to determine the epidemiological and clinical characteristics of COVID-19 in Pakistan. This report will exhibit a linkage between epidemiology and clinical aspects which in turn can be helpful to prevent the transmission of the virus in Pakistan. A retrospective, multiple center study was performed by collecting the data from patients' with their demographics, epidemiological status, history of co-morbid conditions, and clinical manifestations of the disease. The data was collected from 31 public-sector and 2 private hospitals across Pakistan by on-field healthcare workers. A Chi-square test was applied to assess the relationship between categorical data entries. A total of 194 medical records were examined. The median age of these patients was found to be 34 years. A total of 53.6% active cases were present including 41.2% males and 12.4% females till the end of the study. Adults accounted for most of the cases (94.3%) of COVID-19. Fever (86.60%), cough (85.05%), fatigue (36.60%), dyspnea (24.74%), and gastrointestinal discomfort (10.31%) were among the most frequently reported signs and symptoms by the patients. However, 4.12% of the total patient population remained asymptomatic. The median duration of hospital stay was found to be 14 (0–19) days. The earliest source of the spread of the virus may be linked to the foreigners traveling to Pakistan. Spread among men was more as compared to women. A few cases were found to be positive, due to the direct contact with pets or livestock. Hypertension (7.73%), diabetes (4.64%), cardiovascular conditions (2.58%) were the most common co-morbidities. The percentage mortality was 2.50% with the highest mortality among elders.

## Introduction

In December 2019, an event of respiratory disease due to an unknown cause with similarities to that of pneumonia was identified in China (1). Later, the World Health Organization (WHO) acknowledged it to be the sixth emergency service of public health on January 30, 2020 (2) and declared it as a global pandemic in March 2020 (3, 4). On February 11, 2020, the WHO named this viral pneumonia as Corona Virus Disease-19 (COVID-19) (5). The metagenomics analysis was performed through the samples of bronchoalveolar lavage taken from the infected patients (6) and the newly identified pathogen was named as 2019 novel coronavirus (2019-nCoV) by the United States Center for Disease Control and Prevention (CDC) (7). The COVID-19 had almost 88% genetic resemblance to the severe acute respiratory syndrome (SARS). Two SARS viruses were bat-derived coronaviruses bat-SL-CoVZXC21 and bat-SL-CoVZC45 (8). The receptor for the COVID-19 virus is the same as that of SARS-CoV, i.e., angiotensin-converting enzyme-2, ACE-2 receptor (9). The novel corona virus is now listed as the 7th member of the coronavirus family (10).

Multiple epidemiological studies reported that the COVID-19 is identified in Wuhan, China on December 8, 2019 (2, 11–13). This disease later spread worldwide including Iran, Europe, India, United Kingdom (UK), and Pakistan, and officially became a pandemic on March 11, 2020 (13, 14). In Pakistan, the first incidence of this disease was identified at the end of February 2020 (15, 16). COVID-19 is extremely contagious and its spread takes place *via* human-to-human transmissions (17). As of February 15, 2021, the total reported cases in Pakistan were 564,077, while total deaths were around 12,333 and the total recovered were 525,277, as per the data released by the Government of Pakistan (https://covid.gov.pk/).

The coronavirus is encased with an exceptionally huge positive-sense strand of the RNA genome, which mutates very rapidly due to errors in the RNA (10, 18). Pertaining to its continuous mutation, it is highly contagious and may be identified in several animals (19–21). In one of the Indian analysis, the prediction was floated that the cases of COVID-19 will keep on increasing with higher transmission rates as well as with seasonal occurrences (22, 23). Several mathematical models have suggested that the spread of the virus may be retarded by taking precautionary measures including social distancing, isolation, and contact tracing (24, 25). In humans, some patients may remain asymptomatic or may be a carrier of the disease (26–29).

In Pakistan, some patients were reported to be asymptomatic which may serve as a carrier to other people, if not managed properly (30, 31). The purpose of this study is to assess heedfully the epidemiological and clinical characteristics of COVID-19 in Pakistan. This study will exhibit a linkage between epidemiology and clinical aspects which in turn can be helpful to prevent the transmission of the virus in Pakistan.

## Materials and Methods

A retrospective, multiple center study was performed by collecting the patient's demographics, epidemiological status, history of co-morbid conditions, and assessment of clinical manifestations. The data were collected from 33 hospitals (31 public sector hospitals and 2 private sector hospitals) with the help of in-field healthcare workers i.e., doctors, nurses, or pharmacists of the respective hospitals involved in the medical care of these patients. The diagnosis of COVID-19 was made either by taking a specimen from a throat swab and then performing a Real Time-Polymerase Chain Reaction (RT-PCR) in a laboratory setting or by evaluating the clinical symptoms to ascertain the diagnosis. There were 189 lab-confirmed cases; on the other hand, five patients were diagnosed with definitive travel history, signs, and symptoms of COVID-19.

Data collection was initiated on March 16, 2020, and the follow-up of the study was made on April 14th, 2020. Whereas, the data of a few patients were also collected by the end of April 2020. The confirmed 194 cases of the disease through either RT-PCR or clinical diagnosis were considered. A total of 192 cases were confirmed by RT-PCR whereas 2 cases were diagnosed based on clinical manifestations.

Ethical Approval was conferred from Riphah International University, Lahore, Pakistan (Letter No. RCVETS-701). Efforts were made to ensure data collection from different provinces of Pakistan including Punjab, Sindh, Khyber Pakhtunkhwa, Gilgit Baltistan, Islamabad, and Azad Jammu and Kashmir (AJK), a self-governing state under the constitution of Pakistan.

### Statistical Analysis

The data were analyzed by using Statistical Package for Social Sciences version 21 (SPSSv21). Frequency, percentages, median, ranges, and interquartile ranges were used to display data. Mann-Whitney *U*-test was used for comparison across the groups. A Chi-square test was applied to assess the relationship between categorical data entries.

## Results

**Figure 1:**
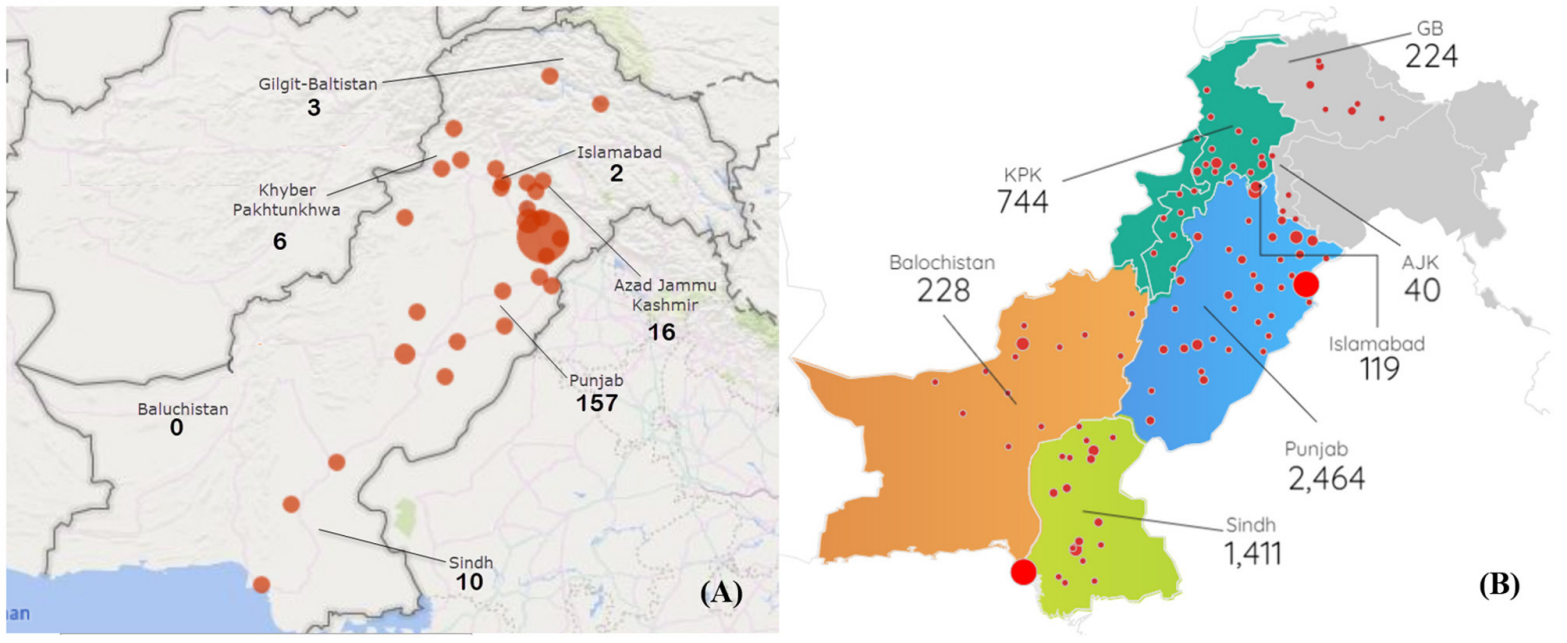
**(A)** Depicts the sample data distribution throughout the country. **(B)** Represents the population data generated by Government of Pakistan. The size red dots represents the number of the patient data collected from each district in **(A)** whereas, the red dots in **(B)** represents the number of active patients. The values below each province name represents the number of patients.

As of April 14, 2020, epidemiological data of 194 patients were collected including 10 (5.15%) patients from the Sindh province, 157 (80.93%) from Punjab, 2 (1.03%) from Islamabad, 3 (1.55%) patients from Gilgit-Baltistan (GB), 6 (3.09%) patients from Khyber Pakhtunkhwa (KPK), and 16 (8.25%) from AJK. The team was unable to collect any data from the province of Baluchistan, the least populated province, due to limited resources. Figure 1A depicts the locations and the amount of patient data collected from that facility. On the other hand, Figure 1B represents the official data by the Government of Pakistan (http://covid.gov.pk/stats/pakistan) of all the patients throughout the country as of April 13, 2020, at 0530 h.

A total of 194 patients' medical records were examined. The median age of these patients was found to be 34 years with an interquartile range (IQR) of 27–48 years. The youngest patient was 6 months old, whereas the oldest one was 87 years of age. Adults accounted for most of the active cases of COVID-19 with 55 (28.4%) patients in 18–29 years of age, 49 (25.3%), 31 (16.0%), and 21 (10.8%) patients were found to be in the age ranges of 30–39, 40–49, and 50–59 years, respectively. The study also included 27 older patients altogether as per locally accepted criterion of old aged individuals. 11 (5.7%) of young patients were also infected.

The distribution of infected males and females were found to be 157 (80.9%) and 37 (19.1%), respectively (Table 1). One of the females was pregnant and tested positive for COVID-19 although, she remained asymptomatic with no reported complications.

**Table 1:** Demographical and epidemiological status of the patients (*N* = 194).

	**All patients** ** (*N* = 194)**	**Male** ** (*n* = 157)**	**Female** ** (*n* = 37)**
**Median age in years (Interquartile range)**	**34 (27–48)**	**33 (27–48)**	**35 (27–50)**
<18	11 (5.7)	7 (4.5)	4 (10.8)
18–29	55 (28.4)	46 (29.3)	9 (24.3)
30–39	49 (25.3)	42 (26.8)	7 (18.9)
40–49	31 (16.0)	24 (15.3)	7 (18.9)
50–59	21 (10.8)	16 (10.2)	5 (13.5)
60–69	20 (10.3)	16 (10.2)	4 (10.8)
≥70	7 (3.6)	6 (3.8)	1 (2.7)
**Gender**			
Female	37 (19.1)		
Male	157 (80.9)		
**Epidemiological status**			
**Local**	**122 (62.9)**	**97 (61.8)**	**25 (67.6)**
Direct contact	34 (17.5)	9 (5.7)	1 (2.7)
Religious congregation	11 (5.7)	11 (7.0)	0 (0.0)
Infected family member	4 (2.1)	0 (0.0)	4 (10.8)
Unknown origin	73 (37.6)	77 (49.0)	20 (54.1)
**Foreign**	**72 (37.1)**	**60 (38.2)**	**12 (32.4)**
Spain	20 (10.3)	17 (10.8)	3 (8.1)
United Kingdom	17 (8.8)	12 (7.6)	5 (13.5)
Iran	10 (5.2)	10 (6.4)	0 (0.0)
Kingdom of Saudi Arabia	7 (3.6)	4 (2.5)	3 (8.1)
Italy	4 (2.1)	4 (2.5)	0 (0.0)
Turkey	4 (2.1)	4 (2.5)	0 (0.0)
Other countries	10 (5.2)	8 (5.1)	1 (2.7)
**Arrival from abroad to hospital admission median time in days (Interquartile range)**	**4 (2–6)**	**4 (2.75–6.25)**	**3 (2–3.75)**
**History of chronic medical conditions**			
Hypertension	15 (7.73)	13 (8.3)	2 (5.4)
Diabetes	9 (4.64)	7 (4.5)	2 (5.4)
Heart conditions	5 (2.58)	5 (3.2)	0 (0.0)
Asthma	3 (1.55)	1 (0.6)	2 (5.4)
Others	5 (2.58)	0 (0.0)	1 (2.7)

*The values represent the number of patients along with percentages [n (%)] where no unit is mentioned. The median age is represented in years along with interquartile range [age (IQR)]. Median time in days along with interquartile range [time (IQR)]*.

The earliest hospital admission of our sample dates was back on February 26, 2020, of a patient who had a recent prior visit to Iran. However, this patient presented to the hospital after 20 days of arrival in Pakistan. Most of the earlier cases were found to be amongst the foreigners. A total of 72 (37.1%) patients had a recent travel history abroad and local transmission comprised 122 patients (62.9%) in this study. Among patients with travel history, 20 (10.3%) patients were from Spain, 17 (8.8%) from the United Kingdom, 10 (5.0%) from Iran, and 7 (3.6%) were from the Kingdom of Saudi Arabia. For most of the patients, the transmission was found to be of unknown origin (37.6%) since they did not have a substantial travel history and were unaware of any contact that could have infected them. 34 (17.5%) individuals had direct contact with the already infected patients of COVID-19. Paramedical staff and doctors are at great risk due to a lack of proper PPE and safety equipment. Among the data collected from different hospitals, most of the patients got infected by direct contact from healthcare providers, including 12 physicians and five paramedical staff. Besides, it was observed that individuals with more public exposure were part of our study, including an epidemiologist, a religious scholar, a lawyer, and a news reporter.

Hypertension was observed to be the most prevalent co-morbidity affecting 15 (7.73%) patients of the total sample. This was followed by diabetes (4.64%), heart conditions (2.58%), asthma (1.55%), and other minor co-morbidities (2.58%) (Table 1).

### Clinical Manifestations

The signs and symptoms of the patients were recorded, and 168 (86.60%) patients exhibited fever. The cough was the second most frequent sign and was experienced by 165 (85.05%) of the patients. 71 (36.60%) patients' complaints of having fatigue. Dyspnea or shortness of breath was the next most occurring symptom (24.74%). Some of the patients also (10.31%) reported gastrointestinal discomfort. 17 (8.76%) patients had the flu, whereas six patients had a cold. Surprisingly, a considerable number of patients (6.70%) also reported a loss of sense of smell and taste. Myalgia, nausea, anorexia, and sore throat were reported by 10 (5.15%), 8 (4.12%), 5 (2.58%), and 3 (1.55%) of patients, respectively. Redness of eyes, dizziness, and anxiety was also observed in 0.52% of the patients. Conversely, 8 (4.12%) patients were asymptomatic. Two patients were put on ventilators; however, both patients expired.

The median duration of hospitalization for COVID-19 patients was found to be 14 days with a stay range of as low as 0 days and as high as 43 days. Paracetamol was the most prescribed medicine (4.64%), followed by chloroquine (1.55%) and cetirizine (1.03%).

Clinical outcomes were evaluated in the last section of data collection. As of April 14, 2020, a total of 70 (36.1%) patients were recovered and discharged. On the other hand, 20 (10.3%) of the deaths were reported. The rest of the patients were still in the hospital, 76 (39.2%) of them were stable and more likely to be discharged in a few days, while 28 (14.4%) patients were still under observation out of which 23 (11.8%) patients recovered and 5 (2.5%) died as per data collected by the end of April 2020. A total of 53.6% active cases were present including 41.2% males and 12.4% females till the end of the study (Table 2).

**Table 2:** Clinical features, signs, symptoms, methods of diagnosis, medications, and outcomes.

**Signs and symptoms**	**All patients** ** (*N* = 194)**	**Male** ** (*n* = 157)**	**Female** ** (*n* = 37)**
Fever	168 (86.60)	136 (86.6)	32 (86.5)
Cough	165 (85.05)	134 (85.4)	31 (83.8)
Fatigue	71 (36.60)	60 (38.2)	11 (29.7)
Shortness of breath	48 (24.74)	35 (22.3)	13 (35.1)
Gastrointestinal discomfort	20 (10.31)	17 (10.8)	3 (8.1)
Flu	17 (8.76)	15 (9.6)	2 (5.4)
Loss of sense of taste and smell	13 (6.70)	10 (6.4)	3 (8.1)
Myalgia	10 (5.15)	7 (4.5)	3 (8.1)
Nausea	8 (4.12)	7 (4.5)	1 (2.7)
Cold	6 (3.09)	6 (3.8)	0 (0.0)
Anorexia	5 (2.58)	4 (2.5)	1 (2.7)
Sore throat	3 (1.55)	3 (1.9)	0 (0.0)
Dizziness	1 (0.52)	1 (0.6)	0 (0.0)
Redness of the eye	1 (0.52)	1 (0.6)	0 (0.0)
Anxiety	1 (0.52)	1 (0.6)	0 (0.0)
Asymptomatic patients	8 (4.12)	7 (4.5)	1 (2.7)
Patients on ventilators	2 (1.03)	1 (0.6)	1 (2.7)
**Methods of diagnosis**			
Specimen by throat swab for RT-PCR laboratory-confirmed	189 (97.4)	154 (98.1)	35 (94.6)
Clinical-confirmed	5 (2.6)	3 (1.9)	2 (5.4)
Median duration of hospital stay in days (range)	14 (0–19)	14 (0–19)	14 (0–15)
**Treatment/medications administered**			
Paracetamol	9 (4.64)	7 (4.5)	2 (5.4)
Chloroquine	3 (1.55)	3 (1.9)	0 (0.0)
Cetirizine	2 (1.03)	2 (1.3)	0 (0.0)
**Clinical outcomes**			
Recovered and discharged	70 (36.1)	64 (40.8)	6 (16.2)
In hospital and stable	76 (39.2)	59 (37.6)	17 (45.9)
In hospital and under observation	28 (14.4)	21 (13.4)	7 (18.9)
Expired	20 (10.3)	13 (8.3)	7 (18.9)

*The values represent the number of patients along with percentages [n (%)] where no unit is mentioned. Median duration of hospital stay in days along with range [duration (Range)]*.

Mann-Whitney *U*-test was applied to evaluate the clinical outcomes of the disease against the patients' age. It was observed that the patients who were recovered had an average age of 33.66 years. The patients who were kept in hospital but were stable had an average age of 37.51 years; those under observation had an average age of 38.61 years. Patients who expired were having an average age of 55.30 years (*P* < 0.05). The highest recovery percentage (72.73%) was among young patients with ages <18 years whereas; the highest mortality percentage was among older patients with an age range of 60–69 years (Table 3).

**Table 3:** Disease outcome status with categorical age-wise distribution.

	**Disease outcome** ***n*** **(%)**			
**Age categories**	**Recovered**	**Stable**	**Under observation**	**Expired**
<18	8 (72.73)	1 (9.09)	2 (18.18)	0 (0.00)
18–29	22 (40.00)	24 (43.64)	7 (12.73)	2 (3.64)
30–39	18 (36.73)	20 (40.82)	9 (18.37)	2 (4.08)
40–49	12 (38.71)	15 (48.39)	3 (9.68)	1 (3.23)
50–59	3 (14.29)	10 (47.62)	3 (14.29)	5 (23.81)
60–69	6 (30.00)	6 (30.00)	2 (10.00)	6 (30.00)
≥70	1 (14.29)	0 (0.00)	2 (28.57)	4 (14.29)

## Discussion

As of April 11, 2020, the COVID-19 attained over 1.6 million cases as per WHO, claiming nearly a hundred thousand lives (32). Recent data presents that COVID-19 cases have been increased and crossed over 80 million globally by February 2021, according to WHO. Pakistan is also amongst the countries affected most by this pandemic with estimated cases of over 0.5 million by February 2021 and a mortality rate of 1.7% (33).

The median age of infected individuals was 34 years. The adult age group (19–59 years) was more affected by the infection. The population demographics of the country, according to the 1998 census, suggesting that nearly 40% of the country's population comprises adults whereas, 53% of the total population is under 19 years of age. 5.54% of the total population is above 60 years of age (34). The occurrence of the infection in females (19.1%) is less as compared to the male (80.9%) population. Our results bear similarity to a recent study in China, where the percentage of the infected females was lesser as compared to the males (26, 35). A previous study also suggested that male mice were more susceptible to the SARS-CoV and MERS-CoV as compared to the female mice (36). Currently, there is no reliable evidence regarding the influence of sex on the susceptibility of the infection. Hence, further studies are required to ascertain this behavior.

The earliest source of the spread of the virus may be linked to the foreigners entering Pakistan from Iran. The disease outbreak in Iran was reported in late January 2020, but the first cases of COVID-19 were identified in late February 2020 (37). Therefore, the dissemination of the virus in Pakistan may be firstly linked to Iran. The travelers from Spain contributed to the highest number of infected patients. According to our data, the local transmission of the virus was massive in the province of Punjab, which is one of the most populated provinces of Pakistan (38). There is already strong evidence of human-to-human transmission of the disease (39). Our study also confirms that individuals with more public exposure are at a higher risk of acquiring the disease. Furthermore, the religious congregations held in March also led to an increased number of cases. Therefore, social distancing must be encouraged to avoid the exponential dissemination of the disease (40, 41).

Another alarming situation observed in our study was the fact that 17 (8.76%) healthcare workers including physicians and paramedics were found to be infected (42, 43). They were also affected by stress and anxiety during the pandemic (44, 45). During the recent coronavirus outbreak in China, a substantial number of healthcare workers acquired the infection (43, 46). A study from China reported that 3.8% of healthcare workers were affected by the disease (47). Another publication discussed the mortality of 23 healthcare workers along with two physicians in China bringing in light the risk these health workers deal with within their daily routine (48). However, in our study, this percentage is quite higher as compared to the reported studies. The lack of personal protective equipment (PPE), prolonged exposure to patients, and inadequate knowledge of the disease transmission among the healthcare providers may have increased such incidents (49, 50). Increased awareness of self-protection, adequate supplies of PPE, and a prompt response may aid in decreased susceptibility of infection among healthcare workers (46, 51, 52).

The most prevalent comorbidities in our study were hypertension, diabetes, cardiovascular conditions, and asthma. A similar pattern was also found in different studies where hypertension was the most prevalent co-morbidity followed by diabetes, heart diseases, and respiratory illnesses (53–55). Some other studies revealed the same pattern as mentioned earlier, with hypertension, diabetes, and cardiovascular diseases as the prime co-morbidities (56, 57).

Fever, cough, fatigue, dyspnea, and gastrointestinal discomfort were among the most frequently reported signs and symptoms by the patients (58). The clinical symptoms of 100 patients admitted to a hospital in Karachi included dry cough, fever, lethargy, fatigue, dyspnea, myalgia, vomiting, nausea, and diarrhea (59). A single-center study from Pakistan has depicted a similar trend of clinical symptoms as our study (60). Other studies have reported a similar set of signs and symptoms (61–63). However, 4.12% of the total patient population remained asymptomatic. This trend is also like the other studies (62, 64, 65). Surprisingly, 6.70% of the patients reported a loss of sense of smell and taste which along with other symptoms, is a strong predictor of COVID-19 infection (66–69). Moreover, flu, myalgia, nausea, cold, anorexia, sore throat, dizziness, redness of the eyes, and anxiety were also reported (45, 63, 70).

Paracetamol, chloroquine, and cetirizine were the most frequently prescribed medicines during the early days of the COVID-19 pandemic in Pakistan (71–74). However, the current data is insufficient to assess the effect of medications on the outcome of the disease. Paracetamol was the most prescribed medicine as it is the safest drug for managing the COVID-19 symptoms in place of ibuprofen (75), followed by chloroquine which is being imagined as a miracle drug (76). Altogether, 20 deaths were reported in our study 104 (53.61%) patients were still hospitalized, with 76 patients in stable condition, and the rest 28 patients were still under observation. Complete recovered patients 70 (36.1%) and were discharged from the hospital. Out of 28 patients, 23 (11.8%) patients recovered and 5 (2.5%) died by the end of April 2020.

There might be a link between COVID-19 with the human population and animals. Some zoo animals were also reported positive for SARS-CoV-2, however, under experimental conditions, chicken and ducks were not affected with COVID-19 (77). Inter-specie transmission of COVID-19 was very recent and must be addressed after conducting different research studies. Different experimental trials suggest that pets (cats, dogs) might also be susceptible to SARS-CoV-2 from humans (19, 78).

The present study has some limitations since 53.61% of the sample patients were still hospitalized, and the recovery of these patients was not ascertained. The data collected was not evenly distributed throughout the country. Moreover, remained unable to investigate more clinical indicators such as complete blood count, CT scans, or chest X-rays since there were a limited number of tests performed by the hospitals. The non-availability of data such as the date of onset of symptoms had prevented us from evaluating more factors such as the incubation period of the virus. The current study is amongst one of the first studies to portray the epidemiological picture of COVID-19 in Pakistan. Being a lower-middle-income country, Pakistan is facing many challenges from inadequate health facilities to poor socioeconomic conditions. Our study may help in identifying and developing a response that may alleviate the rapid onset of disease.

## Conclusions

The earliest source of the spread of the virus may be linked to the foreigners traveling Pakistan. Spread among men was more as compared to females. Fever, cough, fatigue, dyspnea are the most common symptoms. A few positive cases were found to be directly in contact with pets or livestock. Hypertension, diabetes, cardiovascular conditions are the most common co-morbidities. The percentage mortality was 2.50% with the highest mortality among elders.

## Data Availability Statement

The raw data supporting the conclusions of this article will be made available by the authors, without undue reservation.

## Ethics Statement

The studies involving human participants were reviewed and approved by Institutional Ethics Committee (IEC) of Riphah College of Veterinary Sciences. Written informed consent to participate in this study was provided by the participants' legal guardian/next of kin.

## Author Contributions

MA and BB conceived the idea and did a write-up of the manuscript. SR did the critical appraisal of findings with literature search. SA, AM, and RM did the acquisition of patient data and drafting of the article. SM did the analysis and interpretation of the data. MH and MR did general supervision of the research group and final approval of the version to be published. All authors contributed to the article and approved the submitted version.

## Conflict of Interest

The authors declare that the research was conducted in the absence of any commercial or financial relationships that could be construed as a potential conflict of interest.
